# Serum podocalyxin levels correlate with carotid intima media thickness, implicating its role as a novel biomarker for atherosclerosis

**DOI:** 10.1038/s41598-017-18647-6

**Published:** 2018-01-10

**Authors:** Mayumi Shoji, Minoru Takemoto, Kazuki Kobayashi, Toshihiro Shoji, Satoka Mori, Jun-ichi Sagara, Hiroyuki Kurosawa, Yoshiaki Hirayama, Kenichi Sakamoto, Takahiro Ishikawa, Masaya Koshizaka, Yoshiro Maezawa, Koutaro Yokote

**Affiliations:** 1 Department of Clinical Cell Biology and Medicine, Chiba University Graduate School of Medicine, 1-8-1 Inohana, Chuo-ku, Chiba-shi, Chiba 260-8670 Japan; 2 Department of Medicine, Division of Diabetes, Metabolism and Endocrinology, Chiba University Hospital, 1-8-1 Inohana, Chuo-ku, Chiba-shi, Chiba 260-8670 Japan; 3 School of Medicine, International University of Health and Welfare, Department of Diabetes, Metabolism and Endocrinology, 4-3 Kozunomori, Narita-shi, Chiba 286-8686 Japan; 4 Asahi Chuo Hospital, 1326, Ino, Asahi-shi, Chiba 289-2511 Japan; 5 Department of Cardiology, Chiba Emergency Medical Center, Chiba, 3-32-1, Isobe, Mihama-ku, Chiba-shi, Chiba 261-0012 Japan; 6 Life Inovation Research Institute, Denka Co., Ltd, 3-5-1, Asahi-Machi, Machida-City, Tokyo 194-0023 Japan; 7 R&D Center, Denka Seikne Co., Ltd, 1359-1, Kagamida, Kigoshi, Gosen-City, Niigata 959-1695 Japan

## Abstract

Podocalyxin is a cell surface sialomucin, which is expressed in not only glomerular podocytes but also vascular endothelial cells. Urinary podocalyxin is used as a marker for glomerular disease. However, there are no reports describing serum podocalyxin (s-Podxl) levels. Therefore, the association between s-Podxl levels and clinical parameters were examined with 52 patients. s-Podxl level was evaluated using enzyme-linked immunosorbent assay. The median s-Podxl level was 14.2 ng/dL (interquartile range: 10.8–22.2 ng/dL). There were significant correlations (correlation coefficient: r > 0.2) of s-Podxl levels with carotid intima media thickness (IMT) (r = 0.30, p = 0.0307). Multiple logistic regression analysis showed that s-Podxl levels remained significantly associated with carotid IMT > 1 mm (OR: 1.15; 95% CI 1.02–1.31, p = 0.026) after adjustments for traditional cardiovascular risk factors such as age, sex, current smoking status, hypertension, dyslipidemias, and diabetes. In conclusion, s-Podxl is independently associated with carotid IMT and might be used as a novel biomarker for cardiovascular disease.

## Introduction

Cardiovascular disease (CVD), including myocardial infarction and angina pectoris, is a major cause of death worldwide and poses enormous medical and economic burden for most industrialized countries^1^. CVD results from the development of atherosclerosis. To prevent CVD, it is important to identify individuals at a high risk before the onset of CVD. Biomarkers that can predict atherosclerosis at an early stage may facilitate early treatment and reduce CVD-related morbidity and mortality.

Podocalyxin (Podxl) is a cell surface sialomucin, closely related to CD34. It was originally identified as a sialylated protein that was expressed within the kidney glomerular epithelial cell (podocyte) as a glycocalyx^2^. Podxl maintains the podocytes’ shape and slit diaphragm^3,4^. Podxl is expressed outside the kidney within vascular endothelial cells^5^, megakaryocytes^6^, hematopoietic stem cells^7^, and mesothelial cells that line organs^3^ and neurons^8^.

Podxl can be detected in urine that originates from the microvilli or vesicle-like structures when the podocytes are injured^9^. Urinary Podxl is a biomarker for glomerular diseases and used as an early marker for diabetic nephropathy^10,11^. Because Podxl is also expressed in vascular endothelial cells, we hypothesized that Podxl detected in the blood stream was related to vascular injury.

For this purpose, we analyzed serum Podxl (s-Podxl) from 183 patients and examined the relationships between s-Podxl concentration and various clinical parameters, including flow-mediated dilation (FMD), brachial–ankle pulse wave velocity (baPWV), and carotid intima–media thickness (IMT).

## Results

### s-Podxl concentration was significantly correlated with IMT

Basic characteristics of the patients are shown in Table 1. The mean age of the patients was 63 years; 40.4% of the patients were men, 19.2% were current smokers, 65.4% had hypertension, 53.9% had dyslipidemia, and 51.9% had diabetes. Approximately 40% of the patients were receiving statin therapy and 17% were receiving insulin therapy. The mean BMI of the patients was 27.7 kg/m^2^, and the mean HbA1c was 7.5%. The median s-Podxl concentration was 14.2 ng/dL (range, 2.66–37.09 ng/dL).

**Table 1:** Baseline clinical characteristics.

	n	Mean ± SD, n(%)	Min-max
Age (yrs)	52	63 ± 11	25–81
Male	52	21 (40.4%)	—
BMI (kg/m^2^)	52	27.7 ± 8.1	15–57
Current smoker	52	10 (19.2%)	—
Hypertension	52	34 (65.4%)	—
sBP (mmHg)	52	132.3 ± 15.8	91–162
dBP (mmHg)	52	80.3 ± 12.4	55–104
Dyslipidemia	52	28 (53.9%)	—
Use of statin	52	21 (40.4%)	—
LDL-C (mg/dL)	52	106.1 ± 34.6	21–182
HDL-C (mg/dL)	52	51.5 ± 15.3	30–103
TG (mg/dL)	52	115.0 (87.5,195.5)	55–360
Diabetic mellitus	52	27 (51.9%)	—
HbA1c (%)	52	7.5 ± 2.5	5–14.7
CPR index (before meals)	52	1.88 ± 1.44	0.00–7.88
Use of insulin	52	9 (17.3%)	—
eGFR (ml/min/1.73 m^2^)	52	85.41 ± 25.92	33–184
hs-CRP (ng/mL)	52	713.5 (194,1665)	50–18900
ABI	52	1.16 ± 0.10	0.93–1.40
baPWV (cm/s)	52	1655 ± 365	1060–2565
IMT (mm)	52	1.40 ± 0.68	0.5–3.6
FMD (%)	39	7.25 ± 6.00	0.7–24.7
s-Podxl (ng/mL)	52	14.23(10.76, 22.15)	2.66–37.09

Continuous parametric variables were expressed as mean ± SD, and nonparametric variables were expressed as median (IQR). BMI: body mass index, sBP: systolic blood pressure, dBP: diastolic blood pressure, LDL-C: low density lipoprotein-cholesterol, HDL-C: high density lipoprotein-cholesterol, TG: triglycerol, eGFR: estimated glomerular filtration rate, hs-CRP: high-sensitivity C-reactive protein, ABI: ankle–brachial pressure index, baPWV: brachial artery pulse wave velocity, IMT: intima media thickness, FMD: flow-mediated vasodilatation, s-Podxl: serum podocalyxin.

**Figure 1:**
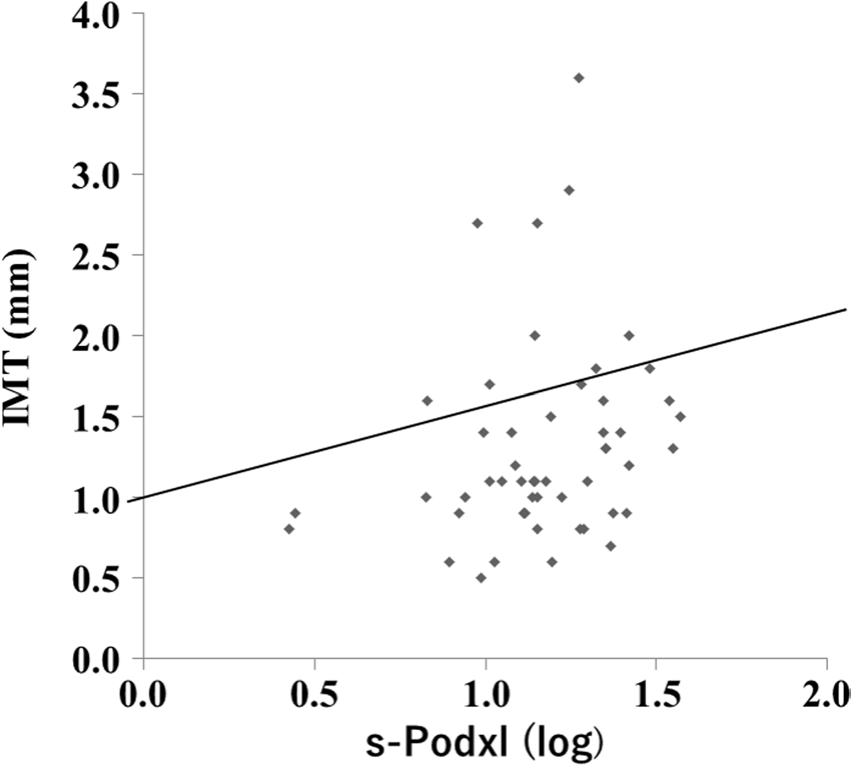
Correlation between s-Podxl concentration and carotid IMT. r = 0.30, p = 0.0307.

Univariate analysis revealed that s-Podxl concentration was correlated (correlation coefficient: r > 0.2) with pulse pressure (r = 0.28, p = 0.0420) and carotid IMT (r = 0.30, p = 0.0307), as shown in Table 2 and Fig. 1. Next, univariate logistic regression analysis showed that sex (OR, 5.16; 95% CI, 1.41–18.91, p = 0.013), HbA1c (OR, 1.34; 95% CI, 1.01–1.80; p = 0.045), and CPR index before meals (OR, 0.57; 95% CI, 0.35–0.95; p = 0.030) and s-Podxl concentration (OR, 1.11; 95% CI, 1.02–1.22; p = 0.023) predicted IMT markers >1 mm, as shown in Table 3.

**Table 2:** Correlations between s-Podxl concentration and other clinical parameters.

	n	r	*p*
Age (yrs)	52	−0.10	0.4958
BMI (kg/m^2^)	52	−0.13	0.3425
sBP (mmHg)	52	0.12	0.4150
dBP (mmHg)	52	−0.10	0.4986
Pulse pressure (mmHg)	52	0.28	0.0420
LDL-C (mg/dL)	52	−0.05	0.7214
HDL-C (mg/dL)	52	−0.36	0.0087
TG (mg/dL)	52	0.17	0.2200
HbA1c (%)	52	0.19	0.1800
CPR index (before meals)	52	−0.17	0.2236
ABI	52	−0.17	0.2304
baPWV (cm/s)	52	0.16	0.2438
FMD (%)	39	0.20	0.2109
IMT (mm)	52	0.30	0.0307
eGFR (ml/min/1.73 m^2^)	52	0.18	0.2013
EPA/AA	52	−0.25	0.0700
hs-CRP (ng/dL)	52	0.10	0.5003
MDA-LDL (mg/dL)	52	0.11	0.4344

(Spearman’s rank correlation coefficient). EPA/AA: eicosapentaenoic acid to arachidonic acid ratio, mdaLDL: malondialdehyde modified Low density lipoprotein.

**Table 3:** Univariate logistic regression analysis for risk of IMT.

Variable	Odds ratio (95% CI)	*p*
Male	5.16 (1.41–18.91)	0.013
Age (per year)	1.04 (1.00–1.08)	0.084
Current Smoker	1.02 (0.25–4.17)	0.978
Hypertension	1.29 (0.41–4.12)	0.665
sBP	1.01 (0.98–1.05)	0.477
dBP	0.98 (0.93–1.03)	0.370
Pulse pressure	1.04 (0.99–1.09)	0.093
Dyslipidemia	2.11 (0.68–6.51)	0.194
LDL-C	1.00 (0.99–1.02)	0.632
HDL-C	0.97 (0.93–1.01)	0.088
TG	1.01 (1.00–1.02)	0.073
Diabetes mellitus	1.85 (0.60–5.66)	0.284
HbA1c	1.34 (1.01–1.80)	0.045
CPR index (before meals)	0.57 (0.35–0.95)	0.030
ABI	18.4 (0.04–8412)	0.351
baPWV	1.00 (1.00–1.00)	0.201
hs-CRP	1.00 (1.00–1.00)	0.119
MDA-LDL	1.01 (1.00–1.02)	0.258
s-Podxl	1.11 (1.02–1.22)	0.023

**Figure 2:**
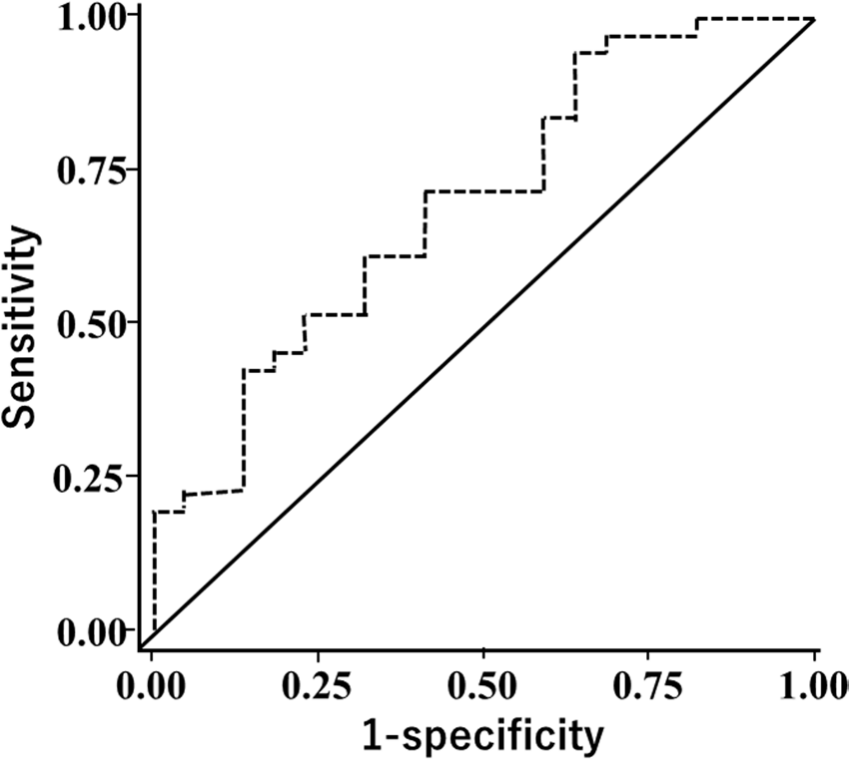
Receiver operating curve (ROC) analysis for IMT > 1 mm. Area under the curve, 0.68; sensitivity, 61%; and specificity, 67%.

With the stepwise logistic regression method, the s-Podxl concentrations remained significantly predictive of IMT > 1 mm (OR, 1.15; 95% CI, 1.02–1.22; p = 0.026) after adjustments for traditional CVD risk factors, such as age, sex, current smoking status, hypertension, dyslipidemia, and diabetes (Table 4). Receiver operating curve (ROC) analysis revealed that s-Podxl concentration of 14.22 ng/dL was the best cut-off value for carotid IMT > 1 mm (area under the curve, 0.68; sensitivity, 61%; and specificity, 67%), as shown in Fig. 2.

**Table 4:** Multivariate logistic regression analysis for risk of IMT.

Variable	OR (95%CI)	*p*
Sex	5.85 (1.30–26.4)	0.022
Age(per year)	1.06 (1.01–1.12)	0.026
s-Podxl (ng/mL)	1.15 (1.02–1.31)	0.026
AIC = 60.65		
AUC = 0.81		
Pseudo R^2^ = 0.25		

Explanatory variables were age, sex, smoking status, hypertension, DL, DM, and s-Podxl.

## Discussion

This is the first report that describes measurements of s-Podxl concentrations. We mainly found that s-Podxl concentration was significantly correlated with IMT and remained independently predictive of IMT > 1 mm, even after adjusting for traditional CVD risk factors, such as age, sex, current smoker, hypertension, dyslipidemia, and diabetes.

CVD prevalence has been increasing worldwide. CVD is caused by atherosclerosis and is a leading cause of death. Therefore, it is important to find biomarkers that can detect early-stage atherosclerosis. Thickening of the inner layer of the arterial wall is evident early in the course of atherosclerotic development. We believe the carotid artery can serve as a “window” for detecting arterial wall thickening. A number of reports indicate that IMT predicts ischemic cardiovascular events^12,13^. Measuring IMT has several advantages, such as low cost and non-invasive measurement. IMT also has disadvantages. IMT changes per year are too small to evaluate the efficacy of anti-CVD therapy and may not be reproducible, depending on how IMT is measured. Biomarkers that relate to IMT may therefore be useful.

Podxl is a type I membrane sialoprotein that belongs to the CD34 family and was originally identified in glomerular podocytes^2^. Podxl is also expressed in a wide variety cells and tissues, including vascular endothelial cells, hematopoietic stem cells, megakaryocytes, platelets, lungs, neurons, and in several kinds of cancers^14^. Podxl has diverse functions, including control of cell adhesion^15^, migration^16^, and cell polarity^17^. Podxl also serves as a ligand for L-selectin^18^ and reorganizes cytoskeletal networks that are mediated by GTPases^19^. Gene deletion of Podxl in mice caused perinatal death because of anuria^3^. This indicates that Podxl is important for opening urinary spaces between podocytes’ foot processes.

Podxl is first translated as a 45-kDa protein. After heavy sulfation and sialylation occur, the mature form of Podxl emerges as a 140–200-kDa protein^20^. Podxl can be found in urine and is used as a marker for glomerular diseases, such as IgA nephropathy^21^ and membranous nephropathy^22^. Podxl is also a novel biomarker for diabetic nephropathy^10,11^.

This study focused on s-Podxl because Podxl is expressed on vascular endothelial cells. The *in vivo* functions of Podxl in vascular endothelial cells have been investigated. Debruin *et al*. reported that endothelial cell-specific deletion of the Podxl gene in mice produced increased lung volume, basal inflammation, vascular permeability, and accumulation of matrix in the lung. Podxl-deleted endothelial cells demonstrate impaired ability to spread over a laminin-coated dish^23^. Horrillo *et al*. reported that conditional knock out of the Podxl gene in endothelial cells in murine increased CRP levels and non-specific inflammatory infiltrates within the vessels (i.e., vasculitis)^24^. Podxl-deleted endothelial cells exhibit delayed recovery of VE-cadherin cell contacts and persistent F-actin stress fibers after thrombin stimulation. All together, these reports indicated that Podxl is important not only for endothelial cell adhesion and barrier stabilization but also for reducing inflammation. Because Podxl is detected in urine, we expected to find Podxl in serum. Indeed, we found Podxl in serum and determined that its levels are correlated with IMT.

It has been reported that Podxl is released from injured podocytes as a consequence of shedding microvilli and/or as vesicle-like structures^25^. We used sandwich-type ELISA with anti-Podxl monoclonal antibodies. These recognized intracellular Podxl used for urinary Podxl. Therefore, s-Podxl could be released from endothelial cells in a similar manner as urinary Podxl.

The kinds of stimulation that increased s-Podxl concentration remain unclear. Endothelial dysfunction initiates atherosclerosis, promoting chronic persistent inflammation^26^. However, we did not observe a relationship between s-Podxl and FMD. This indicates early endothelial dysfunction nor inflammation marker such as high-sensitivity CRP do not influence the expression of s-Podxl. Further investigation is needed to determine how s-Podxl concentration increases in serum in conjunction with IMT thickening.

There are several biomarkers related to CVD, including hs-CRP^27^, fibrinogen^28^, growth-differentiation factor-15 (related to inflammation)^29^, myeloperoxidase^30^, matrix metalloproteinase (seems related to atherosclerotic plaque instability)^31^, lipoprotein-associated phospholipase A2^32^, and soluble CD40 ligand (reportedly relates with platelet activation)^33^. Each of these markers relates to some aspect of atherosclerotic development.

A causal relationship exists between chronic kidney disease and atherosclerosis. This is referred to as cardiorenal syndrome^34^. Because urinary Podxl relates to podocyte injuries and s-Podxl is correlated with IMT, Podxl may be a good biomarker for cardiorenal syndrome.

We have not yet measured urinary Podxl and s-Podxl from the same individual. Therefore, we do not have data to support our hypothesis. However, we have begun to examine the relationships between urinary and serum Podxl and will report on these relationships in the future.

There are several limitations of our study. First, the number of participants was relatively small. Although the ROC analysis was significant, the sensitivity and specificity were low. Therefore, we need to increase the number of patients in future studies to confirm our findings. Second, not only healthy individuals but also the patients with CVD were not included in our study. Third, we were unable to confirm that s-Podxl arise from endothelial cells and not platelet or other cell types.

In conclusion, we can detect Podxl in serum, and s-Podxl concentrations significantly correlate with IMT and remained independently predictive of IMT > 1 mm after adjusted by traditional CVD risk factors. While several studies have shown associations between carotid IMT and future CVD, recommendations regarding the use of carotid IMT for CVD risk prediction are conflicting. Although Podxl is related to IMT, we are not able to conclude that s-Podxl can predict CVD. Therefore, long-term, large-scale evaluations of s-Podxl as a marker for CVD are needed in the future. We believe that s-Podxl, alone or combination with other biomarkers for CVD, will allow us to identify individuals at a high risk for CVD, hopefully preventing future CVD-related deaths.

## Materials and Methods

### Subjects

This was a retrospective, cross-sectional study. All study-related procedures were approved by the Ethics Committee of Chiba University Graduate School of Medicine. We obtained written and signed informed consent from all participants. Initially, 183 patients who were admitted to our Department of Diabetes, Metabolism and Endocrinology at Chiba University Hospital between April 2014 and May 2015 were considered for the study. Among 183 patients, 52 fulfilled the following inclusion and exclusion criteria of our study. All methods were performed in accordance with the guidelines and regulations of Chiba University Graduate School of Medicine.

The inclusion criteria were as follows: patients aged >20 years, those with no history of CVD, and those in whom %FMD, baPWV, and IMT could be measured. Patients were excluded if they had an active infection [C-reactive protein (CRP) level, >5 mg/dL], severe anemia (hemoglobin level, <8 g/dL), or severe hypertriglyceridemia [triglyceride (TG) level, >400 mg/dL] or if they were undergoing hemodialysis.

### Laboratory Measurements

We collected blood samples after an overnight fast from all patients to assess serum glucose, C-peptide immunoreactivity (CPR), total cholesterol (TC), high density lipoprotein (HDL) cholesterol (HDL-C), and TG levels. The CPR index was calculated as follows: (fasting CPR/fasting blood glucose) × 100. Low density lipoprotein (LDL)-C levels were calculated using the Friedewald equation: LDL-C = TC − [(TG/5) × (HDL-C)]. The eicosapentaenoic acid to arachidonic acid (EPA/AA) ratio, malondialdehyde modified (MDA)-LDL level, and high-sensitive CRP (hs-CRP) level were determined by a commercial laboratory (SRL, Inc, Japan).

### Measurements of serum podocalyxin (s-Podxl)

s-Podxl concentrations were measured by Denka Seiken. All serum samples were incubated at 56 °C for 30 min. Then, s-Podxl concentrations were measured using sandwich-type enzyme-linked immunosorbent assay (ELISA). The protein G-bound fraction from ascitic fluid was used as the capture antibody for ELISA plates and was labeled with horseradish peroxidase. Two monoclonal antibodies, which recognized the intracellular peptide region of Podxl, were used for ELISA. The serum was diluted with PBS, and Triton-X was then added to 0.2% (vol./vol.) of the final concentration. ELISA was performed with 100-μl-treated serum samples. We obtained informed consent for the measurement of s-Podxl from all participants.

### Measurement of intima media thickness (IMT)

Carotid IMT was assessed using ultrasound (Aplio 500, Toshiba Co. Ltd., Japan). The patients were examined in the supine position, with the neck rotated in the opposite direction of the probe. Each carotid wall and segment was examined to identify IMT using high-resolution B-mode ultrasonography, with a 7.5 MHz liner array probe. IMT was defined as the distance between the echogenic line, representing the intima–blood interface, and the outer echogenic line, representing the adventitia junction. We evaluated IMT for the common carotid artery, bulbus, and internal carotid arteries on both the right and left side. In addition, we used the maximum IMT from those measurements as representative IMT data for the patients. IMT measurements were obtained by manual ultrasonography performed by two specialists in carotid echocardiography. We obtained informed consent for the measurement of IMT from all participants.

### Measurement of brachial artery flow-mediated vasodilatation (FMD)

The brachial artery FMD was measured in the supine position using UnexEF (Unex Co. Ltd., Japan). Using the echo-probe positioned with a probe holder, a longitudinal image of the right branchial artery was obtained. Then, the forearm-cuff was inflated for 5 min to 50 mmHg above the systolic blood pressure measured before FMD measurement. Following cuff deflation, the diastolic diameter of the branchial artery was semi-automatically recorded for 2 min. The FMD estimate was the percent change in the vessel diameter between the baseline and maximal dilation during the 2 min that followed cuff deflation. FMD was evaluated with the patients in a fasting state.

### Measurement of brachial artery pulse wave velocity (baPWV)

baPWV was measured in the supine position using a vascular testing device (HBP-RPE3-SP form, Fukuda Colin Co. Ltd., Japan). The four cuffs of the volume-plethysmographic device fitted with oscillometric sensors were wrapped around the upper arms and ankles and automatically inflated. The mean of the right and left baPWV values were used for analysis.

### Definition of hypertension

Hypertension was diagnosed according to the Japanese Society of Hypertension Guidelines for the Management of Hypertension 2014. Hypertension was diagnosed when the systolic pressure was ≥140 mmHg and/or the diastolic pressure was ≥90 mmHg or the use of anti-hypertensive agents.

### Definition of diabetes mellitus

Diabetes mellitus was diagnosed according to the Japan Diabetes Society criteria [fasting blood glucose, >126 mg/dl; blood glucose, >200 mg/dl 2 h after the administration of 75 g glucose in an oral glucose tolerance test; and glycosylated hemoglobin (HbA1c), ≥6.5% (National Glycohemoglobin Standardization Program; NGSP)] or the use of oral hypoglycemic anti-diabetic medications.

### Definition of dyslipidemia

Dyslipidemia was diagnosed according to the Japan Atherosclerosis Society Guidelines for the Diagnosis and Prevention of Atherosclerotic Cardiovascular Diseases in Japan-2012 version. Hyper LDL-C was diagnosed when the LDL-C was ≥140 mg/dL; hyper TG was diagnosed when the TG was ≥150 mg/dL; and low HDL-C was diagnosed when the HDL-C was <40 mg/dL.

### Statistical analysis

All data were analyzed using STATA 14.1 software (STATA Corp., College Station, Texas, USA). Parametric continuous values were expressed as mean ± SD, and nonparametric variables were expressed as median (IQR). Categorical variables were reported as percentages. Spearman’s correlation test was used to analyze the correlations between s-Podxl concentration and other clinical parameters. Simple and multiple logistic regression analyses were performed to estimate odds ratios (ORs) and 95% confidence intervals (CIs) between the s-Podxl concentration and IMT > 1 mm. *P* values of < 0.05 were considered to be statistically significant.
